# Homeostasis and the Importance for a Balance Between AKT/mTOR Activity and Intracellular Signaling

**DOI:** 10.2174/092986712801661130

**Published:** 2012-08

**Authors:** DA Altomare, AR Khaled

**Affiliations:** Burnett School of Biomedical Sciences, College of Medicine, University of Central Florida, 6900 Lake Nona Blvd., Orlando, FL 32827, USA

**Keywords:** Signal transduction, growth factor signaling, tumor progression, metabolism, molecular targeted inhibition.

## Abstract

The AKT family of serine threonine kinases is of critical importance with regard to growth factor signaling, cell proliferation, survival and oncogenesis. Engagement of signaling receptors induces the lipid kinase, phosphatidylinositol 3-kinase (PI3K), which enables the activation of AKT. Responsive to the PI3K/AKT pathway is the mammalian target of rapamycin (mTOR), a major effector that is specifically implicated in the regulation of cell growth as a result of nutrient availability and cellular bioenergetics. These kinases mediate the activity of a multitude of intracellular signaling molecules and intersect with multiple pathways that regulate cellular processes. Elucidating the role of AKT/mTOR in metabolism and in hallmark signaling pathways that are aberrantly affected in cancer has provided a solid foundation of discoveries. From this, new research directions are emerging with regard to the role of AKT/mTOR in diabetes and T cell-mediated immunity. As a result, a new perspective is developing in how AKT/mTOR functions within intracellular signaling pathways to maintain cellular homeostasis. An appreciation is emerging that altered equilibrium of AKT/mTOR pathways contributes to disease and malignancy. Such new insights may lead to novel intervention strategies that may be useful to reprogram or reset the balance of intracellular signaling.

## 1 INTRODUCTION

AKT/mTOR signaling has broad effects on cellular signaling with regard to cell proliferation, survival, and oncogenic transformation, while new venues are coming to light that implicate AKT/mTOR signaling in the homeostasis of specific cell types and in certain contexts. mTOR (mammalian target of rapamycin) is an important mediator of the effect of AKT in the contexts of metabolism, diabetes, cellular differentiation (as seen in T-cell mediated immunity), and also aging. Advancing the field is the availability of improved reagents, such as dual inhibitory molecular therapeutics and mouse models to show cooperativity between intracellular signaling molecules. These innovative reagents are facilitating an increased awareness regarding the role of AKT/mTOR and its importance in maintaining the balance within physiologically important intracellular signaling pathways.

The purpose of this review is to highlight the most compelling new discoveries in AKT/mTOR signaling from the perspective of how these protein kinases crosstalk with intracellular signaling pathways to control cell homeostasis. Most investigations to delineate the role of AKT/mTOR signaling in cellular processes have been performed with cultured cells and a limited number of genetically engineered mouse models, although timely studies with transgenic and knockout mice are now becoming more prevalent. These investigations are important to understand the physiological relevance of the AKT/mTOR pathway and its impact on intracellular signaling, since organismal health is often a dynamic interplay between the cellular processes in various tissues or cell types *in vivo*. Our objective is to introduce some of the basic contexts where AKT/mTOR is known to have a major role and then introduce contexts where deregulation of the pathway is known to have a detrimental effect on the health of cells or in *in vivo* models. We will introduce a widely expanding list of molecularly targeted inhibitors/therapeutics that can be used to dissect these pathways and how control of the intracellular signaling cascades has implications for returning cells to homeostatic balance.

A relatively new area of research is booming regarding the role of AKT/mTOR signaling in the proliferation, differentiation, trafficking and survival of hematopoetic cells. Many of the developments evolved as a consequence of the study and understanding of rapamycin as an immunosuppressant, although now it is widely accepted that subsets of immune cells actually may be stimulated or increase in number in response to mTOR inhibition. Since the immune system is composed of a diverse array of hematopoietic cells with different functions, most of the discussions of the literature presented here will highlight the role of AKT/mTOR signaling in the regulation of T cell maturation and activation, with an emphasis on understanding the impact upon effector T cells.

## 2 GENERAL ROLE FOR AKT/MTOR 

### 2.1 Growth Factor Signaling

AKT is a central player in processes downstream of activated growth factor receptor signaling, and has been extensively reviewed elsewhere [1-3]. Examples of growth factor receptor signaling include the insulin receptor, epidermal growth factor receptor and hepatocyte growth factor receptor. The role of AKT in transducing signals mediated by these tyrosine receptor kinases is relatively uniform across different cell types. All three highly conserved AKT isoforms (AKT1, AKT2 and AKT3) are activated by the same mechanism, although they may have slightly different significances in certain tissue-specific contexts, which will be reviewed below. As shown in Fig. (**1**), downstream of ligand binding to tyrosine receptor kinase, AKT recruitment to the plasma membrane is mediated mainly through phosphatidylinositol 3-kinase (PI3K), which phosphorylates phosphoinositides to generate phosphatidylinositol (3,4,5)-trisphosphate (PIP3). The amino terminal pleckstrin homology (PH) domain of AKT binds PIP3, thereby promoting the translocation of AKT to the plasma membrane where it is phosphorylated and activated. The tumor suppressor PTEN acts as a regulator of AKT activity by dephosphorylating PIP3, although it is frequently downregulated or lost during tumor progression, contributing to deregulation of the pathway in cancer cells. Moreover, a recent review [4] cites a diverse group of Ack1/TNK2 [5], Src [6], and PTK6 [7] tyrosine kinases and TBK1[8, 9], IKBKE [10, 11], and DNA-PK [12] serine/threonine kinases that activate AKT directly to promote pro-proliferative signaling functions in response to growth factor stimulation or inflammatory or genotoxic stimuli.

**Fig. (1):**
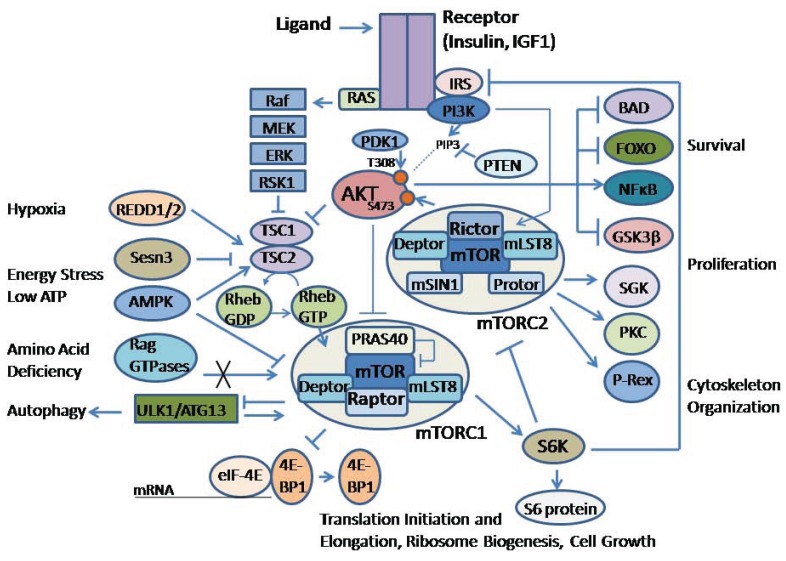
Schematic diagram of the AKT/mTOR signaling pathway. Insulin or insulin growth factor-1 (IGF-1) stimulation is shown to represent growth factor signaling. Upon activation the insulin receptor (IR) phosphorylates insulin receptor substrate (IRS), which activates phosphatidylinositol 3-kinase (PI3K), which in turns phosphorylates phosphoinositides to generate phosphatidylinositol (3,4,5)-trisphosphate (PIP3). Phosphatase and tensin homolog (PTEN) can dephosphorylate PIP3 to regulate activity of the signaling pathway. AKT is activated by binding PIP3 to it the amino terminal pleckstrin homology (PH) domain, which promotes translocation of AKT to the plasma membrane (represented by a dotted line) where the carboxyl terminal Thr308 is phosphorylated by phosphoinositide-dependent kinase-1 (PDK1), and Ser473 is phosphorylated by mTORC2. AKT regulates several cellular processes such as survival and cell proliferation through a variety of downstream proteins, including BAD (Bcl-2-antagonist of cell death), FOXO (Forkhead Box O), NFćB, and GSK-3β (glycogen synthase kinase 3-beta), among others. AKT can directly phosphorylate and inactivate a 40kDa, proline-rich protein (PRAS40), to relieve the inhibitory regulation on mTORC1 activity. AKT also can phosphorylate and inactivate the tuberous sclerosis (TSC) tumor suppressor protein complex, which acts as a GTPase-activating protein for the RAS-related small G protein (Rheb) to regulate its activity. Retention of the Rheb-GTP form activates mTOR. mTOR is complexed with other proteins including Raptor, mLST8 and Deptor in complex I (mTORC1) and Rictor, mLST8, Deptor, Sin1 and Protor in complex II (mTORC2). mTORC1 regulation is mediated through a variety of environmental signals, which are mediated by proteins such as REDD (regulated in development and DNA damage responses 1), Sestrin 3 (Sesn3), AMP-activated protein kinase (AMPK) and Rag GTPases. mTORC1 phosphorylates downstream p70S6 kinase (S6K) and the eukaryotic initiation factor 4E-binding protein (4E-BP1), which releases it from inhibiting eIF4E so that 40S ribosomal subunit can be recruited to the 5’ end of mRNAs to initiate protein translation. S6K phosphorylates ribosomal protein S6, which is also involved in regulating protein translation through the 40S ribosomal subunit. mTORC1 is also instrumental in regulating autophagy through ULK1 and ATG13 (autophagy related 13 homolog). In contrast, mTORC2 regulation is still being investigated, although it is known to be activated by growth factor signaling pathways. mTORC2 phosphorylates a different set of proteins, including serum- and glucocorticoid-inducible kinase (SGK), protein kinase C (PKC) and the guanine nucleotide exchange factor Phosphatidylinositol (3,4,5)-Triphosphate-Dependent Rac Exchanger 1 (P-Rex1) to regulate Rac mediated cytoskeleton changes. Importantly, mTORC2 phosphorylates AKT as a feedback into the pathway. Phosphorylation of S6K downstream of mTORC1 is a negative feedback to inhibit IRS signaling and Rictor phosphorylation. The growth factor mediated activation of the RAS/Raf/MEK/ERK/RSK1 pathway is another mechanism whereby there can be crosstalk regulation of the AKT/mTOR signaling pathway.

Overall, activated AKT kinases phosphorylate numerous substrates that control a variety of downstream processes in both normal and cancer cells (Fig. **1**). In normal cells and several tumor types, insulin receptor (IR) and insulin-like growth factor (IGF) receptor activation are examples of growth factor signaling pathways that transduce effects on cell metabolism, growth and survival, as reviewed previously [13]. In brief, following the binding of ligand to the receptor, tyrosine phosphorylation of IR substrates and Shc initiates canonical signaling through PI3K/AKT and RAS/MAP kinase pathways, which are known to mediate the actions of insulin and IGFs. 

Several effectors in the intracellular signaling pathways are known to have evolutionarily conserved consensus sites that are indicative of being direct substrates of AKT [2]. Among these are Forkhead box protein O1 (FOXO1), glycogen synthase kinase 3-beta (GSK-3β), and a 40kDa, proline-rich protein (PRAS40), which is a regulator of the mTOR complex 1 (mTORC1). Early studies demonstrated that growth factors activate mTORC1 through PI3K activation and phosphorylation of downstream AKT. A complex interplay between AKT and mTOR signaling exists, including several feedback mechanisms to control the phosphorylation of AKT [2]. 

mTOR is a kinase that is a downstream effector of AKT. It is known to be an integral component of two distinct complexes called mTORC1 and mTORC2 (Fig. **1**). The complexes are distinguished through the binding of mTOR to accessory proteins, such as the regulatory associated protein of mTOR (Raptor) found in mTORC1 and rapamycin-insensitive companion of mTOR (Rictor) in mTORC2, as previously reviewed [14]. mTORC1 is regulated through the actions of AKT on PRAS40 and on the tuberous sclerosis (TSC) tumor suppressor protein complex (Fig. **1**). TSC2 is a GTPase-activating protein for the RAS-related small G protein (Rheb), and inactivation of TSC complex through phosphorylation by AKT is needed to favor the GTP-bound Rheb and activation of mTOR. 

mTORC1 substrates are the S6 kinases S6K1 and S6K2, and the 4E-BP1 and 4E-BP2 proteins, which regulate several aspects of mRNA translation, as has been reviewed [15]. In contrast, the regulation of mTORC2 appears to be different from that of mTORC1 and is known, at least in part, to be mediated through growth factor signaling [14]. mTORC2 downstream effectors also are distinct from that of mTORC1 and include AKT, in addition to protein kinase C (PKC), serum- and glucocorticoid-inducible kinase (SGK), and phosphatidylinositol (3,4,5)-triphosphate-dependent Rac exchanger 1 (P-Rex1) mediated Rac signaling. Importantly, phosphorylation of Ser473 by mTORC2, along with phosphorylation of Thr308 by phosphoinositide-dependent kinase-1 (PDK1), is necessary for full activation of AKT.

The S6K1 effector of mTOR is a pivotal downstream effector of mTORC1 that can signal back to inhibit insulin receptor substrate (IRS) (Fig. **1**). Although there are four IRS proteins, studies with transgenic mice suggest that most insulin/IGF signals are mediated through IRS1 and IR, as reviewed previously [16]. IRS1 is associated with glucose homeostasis, whereas IRS2 is linked to the regulation of lipid metabolism [13]. The S6K1 feedback mechanism is known to regulate signaling through the IR receptor, and consequently AKT kinase. Recent papers also have shown that S6K1 can inhibit Rictor protein [17]. This may be a mechanism for keeping the balance between mTORC1 and mTORC2 activity, and indirectly feedback to regulate AKT activity. Although broadly understood, there are many intermediate intracellular signaling molecules that mediate these basic steps and mechanistic details need to be elucidated. 

In general, signaling through growth factor receptor pathways activates the expression of pro-survival and anti-apoptotic genes. AKT mediates cell survival through the phosphorylation and subsequent inactivation/cytoplasmic retention of FOXO transcription factors, the phosphorylation and cytoplasmic sequestering of BAD, and the activation/nuclear translocation of the NF-κB transcription factor through the phosphorylation of IKKα, which in turn phosphorylates IκB, targeting it for proteasomal degradation, as has been extensively reviewed elsewhere [18]. In the absence of growth factor signaling, apoptosis may occur through an intrinsic pathway mediated by the release of cytochrome c and subsequent activation of caspase 9. Alternatively, activation of death receptors (Fas and TNFR) may trigger an extrinsic cell death pathway that activates caspase 8. 

Mechanisms enable a signaling interplay between AKT, mTORC1 and FOXO1. FOXO1 binds to the promoter region of Sestrin3 (Sesn3) to increase its expression [19]. Sestrins are known to accumulate in cells under stress and to activate adenosine monophosphate–activated protein kinase (AMPK). Although the exact mechanism has yet to be delineated, Sesn3 is upstream of TSC2 and therefore regulates the Rheb protein that can activate mTORC1 (Fig. **1**). In another TSC2-independent mechanism, FOXO1 increased the expression of Rictor leading to increased mTORC2 activity and therefore AKT activity [19]. Increased Rictor was associated with decreased mTORC1 activity, perhaps, in part, through decreased mTOR-Raptor levels. Hence, FOXO1 may maintain homeostatic balance between AKT and mTOR activities. The results are indicative of a delicate balance between AKT and mTORC1 activities. 

As stated above, activation of mTORC2 via PI3K may be different and is still being defined. The activity of mTORC2 may be cell context dependent because of its links to processes such as cell migration and glycogen metabolism [14]. Previous reviews have summarized that TORC2/mTORC2 is a mediator of actin cytoskeleton organization and cell polarization, and is known to control cytoskeleton regulators such as Rho1 GDP–GTP exchange protein 2 (Rom2) and the protein kinase A/G/C (AGC) Ypk2 in yeast, and P-Rex1 mediated Rac signaling in cancer cells [20]. mTORC2 also is known to facilitate the accumulation of glycogen by activating AKT, which phosphorylates and inactivates GSK-3β, leading to the activation of glycogen synthase. Sirtuin 1 (Sirt1) also positively regulates expression of Rictor, thereby increasing the phosphorylation of AKT and the inhibition of downstream FOXO1 to downregulate the gluconeogenic genes glucose-6-phosphatase (G6pase) and phosphoenolpyruvate carboxykinase (Pepck) [21]. In this way, when glucose is abundant, insulin signaling through mTORC2/AKT may contribute to decreased expression of gluconeogenic genes to suppress gluconeogenesis in the liver. 

Active mTORC2 also is known to be associated with ribosomes, and insulin-stimulated PI3K signaling has been shown to promote mTORC2-ribosome binding [22]. A genetic screen in yeast and subsequent studies in mammalian cells revealed that ribosomes, but not protein synthesis, are required for TORC2 signaling. Data with melanoma, colon cancer, and HeLa cells, suggest that mTORC2-ribosome binding increases AKT phosphorylation, whereas disruption of the mTORC2-ribosome complex induces apoptosis in PTEN-deficient melanoma cells. Thus, the TORC2-ribosome interaction may be conserved in normal and cancer cells. 

### 2.2 Metabolism

Cell metabolism is a tightly regulated process that is highly dependent on cell context. The general overview presented here describes the role of AKT/mTOR in mediating mitochondrial metabolism through glucose in normal and deregulated pancreatic islet cells, how glycolysis and other energy producing mechanisms are used to support rapid tumor cell growth, and the emerging role of mTORC1 and mTORC2 in mediating signaling in response to metabolic stress that may result from these or other cellular contexts.

Insulin and insulin-like growth factor I (IGF-I) regulate growth and metabolism of several types of mammalian cells, including pancreatic β-cells. Glucose regulates proliferation and survival of β-cells, as well as stimulating insulin secretion. Through exogenous glucose or insulin in β-cell lines lacking either IR or IRS2, it was shown that glucose stimulation of growth and survival of β-cells requires the activation of the insulin signaling pathway [23]. Addition of either insulin or glucose activated insulin signaling in control β-cell lines, with the effects of insulin peaking earlier than glucose. In contrast, insulin stimulation of IR and IRS2 knockout cells resulted in decreased PI3K/AKT activity and glucose stimulation was ineffective to activate PI3K/AKT, and instead resulted in activated extracellular signal-regulated kinase (ERK). 

Insulin has been shown to activate IR and stimulate PI3K/PDK1/AKT and GRB2/SOS/RAS kinase cascades, which are known to regulate mitochondrial function, as reviewed elsewhere [16]. Glucose and lipid metabolism converge in mitochondria, which generate the majority of cellular energy (ATP) by coupling the tricarboxylic acid (TCA) cycle with oxidative phosphorylation (OXPHOS). Disrupted insulin signaling leads to hyperglycemia and dyslipidemia. The deregulation of glucose and lipid metabolism can induce health problems, including obesity, cardiovascular disease and type 2 diabetes when pancreatic β-islet cells fail to secrete enough insulin or under conditions of insulin resistance. Mitochondrial function is impaired during insulin resistance and in metabolic disease. 

**Fig. (2):**
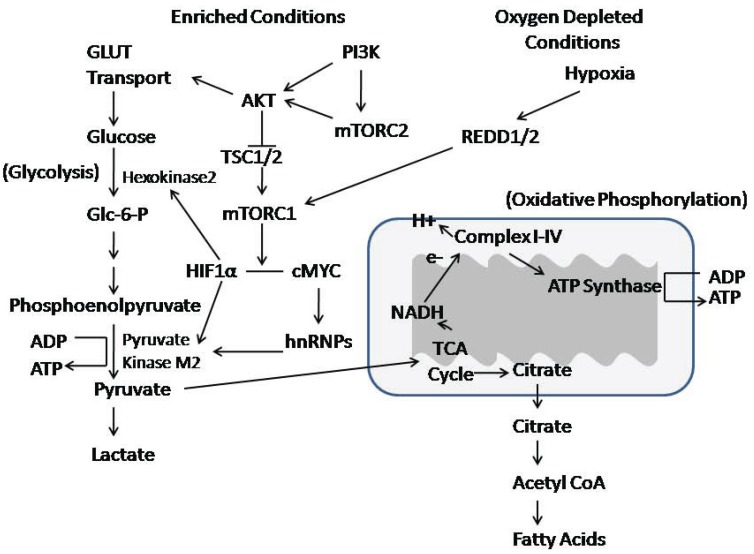
AKT/mTOR signaling and crosstalk with glycolysis and mitochondrial oxidative phosphorylation. Glucose is transported into the cells using GLUT transporters. Glycolysis uses glucose to make pyruvate, which can be converted to lactate, or used to generate ATP through oxidative phosphorylation. Oxidative phosphorylation occurs in the mitochondria. The process converts pyruvate to acetyl coenzyme A (acetyl-CoA), and uses the tricarboxylic-acid (TCA) cycle to produce free electrons (e-) that are carried by NADH to the electron-transport chain, resulting in a movement of protons (H+) out of the mitochondrial matrix to establish a electrochemical potential that cumulates in the activation of ATP synthase to synthesize ATP. AKT/mTOR signaling interacts with these metabolic processes through the regulation of GLUT transport. Moreover, hypoxia-inducible factor 1 alpha (HIF1α) is a downstream effector of mTORC1 that is important in upregulating the expression of hexokinase 2 and pyruvate kinase M2, two key rate-limiting enzymes in the glycolytic pathway. Another mechanism downstream of mTORC2 is mediated by cMYC, which activates heterogeneous nuclear ribonucleoproteins (hnRNPs) in the transcription of pyruvate kinase M2.

Cells under mitochondrial stress develop resistance to apoptosis and develop a shift in cell metabolism, which involves the activation and increased levels of nuclear AKT [24]. AKT mediated phosphorylation of heterogeneous ribonucleoprotein A2 (hnRNPA2), a nuclear RNA-binding protein and a transcriptional coactivator for the mitochondrial stress-target genes was shown to be important for cell survival under mitochondrial respiratory stress (Fig. **2**). The activation of AKT1 and the phosphorylation of hnRNPA2 in response to mitochondrial respiratory stress involved a similar set of proteins (C/EBP, c-Rel/p50, NFAT, and CREB). These results suggest a complex regulatory pathway in which there is crosstalk between AKT signaling and hnRNPA2. 

Additional insights into cell metabolism have been derived from the study of cancer cells. It has been postulated that “waves” of gene expression promote metabolic changes that occur during carcinogenesis [25]. The process is initiated with oncogene-mediated changes, followed by hypoxia-induced factor (HIF)-mediated gene expression. Both set of changes result in a highly glycolytic “Warburg” phenotype and suppression of mitochondrial biogenesis, a process activated during cell stress or in response to environmental stimuli to generate new mitochondria. Metabolic transformation may be initiated by oncogene-mediated signaling such as that of AKT. The second wave is a consequence of hypoxic signaling. There may be restoration of mitochondrial biogenesis in a second oncogene-induced reprogramming stage that involves AKT and MYC, whereby MYC converts cells to glutaminolysis to serve as an alternative pathway for production of ATP. Retrograde signaling from revitalized mitochondria might constitute a fourth wave of gene reprogramming [25].

Although aerobic glycolysis is a hallmark of cancer, it is not understood when, how, and why cancer cells become highly glycolytic. Aerobic conversion of glucose to lactate is characteristic of cancer cell metabolism, and tumor hypoxia may provide a selective pressure that contributes to increased glycolysis, as has been recently summarized [26]. Cancer cells need to adapt so that they can balance the high nutrient demand of rapid proliferation and input from the extracellular environment. Glycolysis helps meet the increased demands for energy production. Elevated glucose metabolism influences transcription and extracellular matrix remodeling which may additionally impact tumorigenicity. AKT is a kinase that has been shown to be involved in the regulation of both glycolytic and oxidative metabolism [27]. 

mTOR also is a critical mediator of aerobic glycolysis, in part through the induction of pyruvate kinase M2 (PKM2) and other glycolytic enzymes [28]. PKM2 expression increased in mouse kidney tumors with loss of TSC2 and activation of mTOR, but decreased in human cancer cells with reduced mTOR activity. The mechanism was mediated by hypoxia-inducible factor 1α (HIF1α) transcriptional upregulation of PKM2 expression and hnRNPs-dependent regulation of PKM2 gene splicing. Disruption of PKM2 suppressed mTOR-mediated tumorigenesis. Unlike normal cells, tumor cells with an elevated mTOR activity were more sensitive to inhibition of mTOR or glycolysis, and dual suppression of mTOR and glycolysis acted synergistically to inhibit proliferation and tumor growth. 

In general, mTORC1 acts as a signal integrator for four major regulatory inputs: nutrients, growth factors, energy and stress (Fig. **1**). mTORC1 regulates growth by maintaining a balance between anabolic vs. catabolic metabolism [15]. In other words, it affects balance between protein synthesis and nutrient availability vs. autophagy and utilization of energy stores. 

Nutrients and amino acids are known to be important regulators of mTORC1 activity. Furthermore, a recent study showed that amino acids may also activate mTORC2 [29]. Amino acids induced a rapid phosphorylation of AKT at Thr308 and Ser473, and only phosphorylation of Ser473 was dependent on Rictor. Kinase assays confirmed mTORC2 activation by amino acids. Although these results need further clarification in future studies, it was shown that under different starvation conditions, amino acids can selectively activate mTORC1 or mTORC2. 

Protein synthesis is energy intensive and requires downstream targets in the AKT/mTOR pathway to function together to control stages of mRNA translation from ribosome biogenesis to translation initiation and elongation, as summarized previously [30]. Deregulation of ribosome biogenesis, translation initiation, and translation elongation are frequently observed in cancer. AKT/mTOR controls protein synthesis by ribosome biogenesis through enhanced rRNA synthesis and enhanced ribosomal protein production, and also through the activation of translation initiation factors that drive cap-dependent translation. The latter process is a rapid means by which AKT can activate protein synthesis through mTORC1-dependent phosphorylation of the 4E-BPs. AKT also affects efficiency through the control of translation elongation factors. In general, AKT regulates protein translation, as well as the translation of specific mRNAs.

### 2.3 Survival Signaling

In addition to mediating growth factor signaling and metabolism in the growth and proliferation of a variety of normal and cancer cell types, the AKT/mTOR pathway is known to facilitate survival and protect against apoptosis. There are numerous reports further beyond the scope of this review regarding the role of AKT/mTOR in survival pathways, and how targeted disruption of metabolic pathways may be used to sensitize cancer cells to apoptosis. Here, mTORC1, is also highlighted with regard to its link to autophagy as a mechanism to prolong cell survival under metabolic stress or in opposition to apoptosis. The manipulation of the autophagic response in cancer cells in order to reprogram them for apoptosis still needs clarification. 

Disruption of aerobic glycolysis may be a strategy to target aberrantly functioning cells. However, the mechanistic links between metabolism and apoptosis are not understood. For example, glucose metabolism requires Bcl-2 family proteins to initiate rapid apoptosis, since cells lacking proapoptotic Bax and Bak can resist cell death in periods of nutrient starvation through autophagy. In the presence of adequate glucose, AKT inhibits decreased expression of the Bcl-2 protein, Mcl-1, and protects cells from growth factor deprivation-induced apoptosis through Mcl-1 association and inhibition of proapoptotic Bim [31]. Disrupted glucose metabolism led to increased Bim, decreased Mcl-1, and apoptosis. Disruption of glucose metabolism or inhibition of mTORC1 overcame Mcl-1–mediated upregulation and resistance to the proapoptotic Bcl-2/Bcl-xL/Bcl-w inhibitor ABT-737 in diffuse large B cell leukemic cells. Collectively, results suggested that glucose metabolism of tumor cells relies on mTOR activity and 4E-BP1 phosphorylation to sustain Mcl-1 protein translation, and that diminished expression of Mcl-1 through the disruption of aerobic glycolysis may sensitize cancer cells to apoptosis.

As extensively reviewed by others, autophagy is an evolutionarily conserved intracellular mechanism to prevent the toxic accumulation of damaged or excess proteins by sequestering organelles and/or proteins into vesicles that are subsequently degraded through fusion with lysosomes [32]. Through this process, cells can release nutrients and essential building blocks for the purpose of recycling cellular components to sustain metabolic homoeostasis. Cancer cells also can use autophagy as a means of resistance in times of metabolic and therapeutic stress to avoid apoptosis. Although these mechanisms still need to be clarified, there is collective evidence that cancer cells may use autophagy to evade mechanisms that regulate cell survival and proliferation [33].

mTORC1 is a major regulator of autophagy. Autophagy is upregulated during periods of starvation or growth factor withdrawal, oxidative stress, infection, or the accumulation of protein aggregates. Downstream of mTORC1 are proteins that are required for autophagy, including Atg1/ULK, which plays an essential role in the formation of the preautophagosome [15]. Autophagy also is upregulated by AMP activated protein kinase (AMPK), which is an energy sensor and regulates cell metabolism to maintain energy homeostasis. Coordinated phosphorylation of Ulk1 by AMPK and mTOR is important for autophagy, and may be a mechanism by which cells can respond to complex extracellular signals to ensure that autophagy is only initiated under severe starvation conditions [34]. 

In MDA-MB231 breast cancer cells treated with AKT isoform-specific siRNAs, prolonged suppression of the AKT2 isoform resulted G0/G1 cell cycle arrest through the downregulation of Cdk2 and cyclin D, and upregulation of p27 [35]. Inhibition of AKT2 was linked to mitochondrial biogenesis, as assessed by an increase in the mitochondrial volume and upregulation of the PGC-1α regulator of mitochondrial biogenesis. Prolonged inhibition of AKT2, but not AKT1 or AKT3, led to cell death by autophagy of the mitochondria (mitophagy). Thus, AKT2 in these breast cancer cells was important in cell cycle progression and protective against autophagy through mitochondrial homeostasis. AKT2 also targeted and activated p70S6K, a downstream target of mTORC1, although additional studies are needed to understand how specific players in the AKT/mTOR signaling pathway play a role in the regulation of autophagy and the switch to apoptosis.

### 2.4 Immune Cell Function

Rapamycin is a small molecule inhibitor that interacts with FKBP12 to bind to the FKBP12–rapamycin-binding (FRB) domain of mTOR, and is known to have immunosuppressant characteristics. The use of rapamycin has revealed functions predominantly associated with mTORC1, although threshold concentrations of the drug are not clearly defined and persistent or high concentrations of rapamycin may affect mTORC2. Importantly, new studies are emerging that AKT/mTOR is involved in immune cell homeostasis and differentiation. There are several recent reviews that address the role of mTOR in immune cell function [36-38]. Overall, it is evident that mTOR has an important role in modulating innate and adaptive immune responses. Recent studies show that some immune cells may be stimulated by mTOR, including certain populations in the CD4 and CD8 subsets of cells that are important for anti-tumor immunity.

There are different types of T cells including helper, cytotoxic, memory, regulatory and natural killer cells. mTOR regulates the function of antigen-presenting cells, such as dendritic cells, and has important roles in the activation of effector T cells, and in the function and proliferation of regulatory T cells, as reviewed previously [36]. In conventional T cells, mTOR integrates the T cell receptor (TCR) and CD28 signals that are necessary for T cell activation. T cell activation requires overcoming checkpoints at the G0 to G1 and G1 to S phases of the cell cycle. The first checkpoint requires the activation of nuclear factor of activated T cells (NFAT), mitogen-activated protein kinases (MAPKs), activator protein 1 (AP1) and nuclear factor-κB (NF-κB), and these players coordinately can regulate the expression of interleukin-2 (IL-2) and its receptor IL-2R. mTOR can complex with survivin and aurora B, in addition to mTORC1/mTORC2, to overcome the second checkpoint in the transition from the G1 to S phase of the cell cycle in T cell activation [36]. Moreover, mTOR is known to regulate the transcription factor Kruppel-like factor 2 (KLF2) that controls the expression of lymphoid tissue-homing molecules. 

The differentiation of naïve T cells into effector cells or memory cells is controlled by antigen receptors, co-receptors, cytokines and chemokines. Emerging evidence suggests that mTOR activity regulates the development of CD4 T cell subsets (Th1, Th2, Th17, and follicular helper T or Tfh) and support the regulatory role of mTOR in the differentiation of T regulatory cells (Tregs) [38]. Treg cells are less metabolically demanding than their effector counterparts, exhibiting decreased mTOR activity but increased Pim2 kinase activity [37]. mTOR activity impacts the ability of Treg cells to proliferate when stimulated in the presence of rapamycin [36]. The mechanism for the preferential expansion of Foxp3 CD4 cells in the absence of mTOR signaling have yet to be fully elucidated. 

**Fig. (3):**
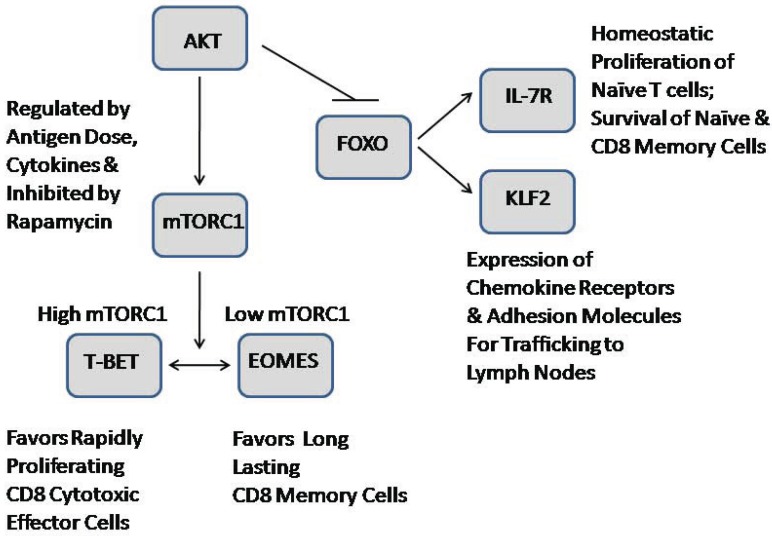
Role of AKT/mTOR signaling in the regulation of T cell differentiation, trafficking and survival. mTORC1 regulates the balance of T-bet and Eomes transcription factors, which are known regulators of effector versus memory CD8 T cell function. T-bet is required for the cytotoxic functions and the production of effector cytokines. Eomes expression is required for the memory. Furthermore, inhibition of FOXO1 by AKT phosphorylation results in the upregulation of the expression of Krüppel-like factor 2 (KFL2) to increase CD8 T cell migration and in the expression of interleukin-7 receptor (IL-7R), which is important for the survival of naïve and memory CD8 cells. Figure based on that of Finlay and Cantrell [39].

Several recent reviews have addressed an emerging role for mTOR in directing T cell activation and differentiation [38, 39] (Fig. **3**). Collectively, mTOR is implicated in the integration of signals from the microenvironment, in the programming of CD4 effector versus regulatory T cells and the generation of CD8 effector versus memory cells, in addition to the role of T cell trafficking and determination of T cell activation [37]. Overall, mTOR is linked to T cell metabolism and function, although the impact of nutrient availability has yet to be defined. Naïve T cells are catabolic and have low levels of mTOR activity, but activation and generation of effector CD4 and CD8 T cells are linked to high metabolic demands and elevated mTOR activity, as recently reviewed [37]. mTOR inhibition by rapamycin in CD8 T cells favors the generation of memory cells, which are characterized by low metabolism, low mTOR activity, and increased expression of the FOXO, KLF2, and eomesodermin (Eomes) transcription factors. Trafficking is dependent on cell surface markers such as CD44, CD62L, and CCR7 for controlling T cell migration of naïve, effector, and memory CD8 T cells to lymphoid organs. During T cell activation, these receptors are downregulated and other receptors are upregulated to facilitate migration to areas of inflammation or infection. AKT/mTOR also may be important from the perspective of metabolism in T cell trafficking, since naïve cells downregulate CD62L and CCR7 as they require more energy. 

Naïve and memory CD8 T cells circulate between the blood, lymphoid tissues and lymphatics, whereas activated CD8 T cells clonally expand in the lymphoid tissue, differentiate into effector cytotoxic T lymphocytes (CTLs) and then migrate to the site of infection or tumor antigen [39]. New findings demonstrate the role of AKT and cytokine signaling in memory or CTLs. Namely, constitutive AKT activation was shown to repress interleukin-7 receptor (IL-7R) and IL-15R expression, signal transducer and activator of transcription-5 (STAT5) phosphorylation and Bcl2 expression, but did not facilitate memory T cell survival. In contrast, constitutive STAT5 increased both effector and memory T cell survival, T cell proliferation, AKT activation and Bcl2 expression [40]. A balance between AKT and STAT5 signaling may be needed for CD8 T cell viability and survival. Regarding TCR – IL-2 signaling in CTLs, AKT may control transcription of effector molecules (e.g., adhesion molecules and cytokine and chemokine receptors) that distinguish memory from naïve T cells [41]. AKT may not be needed for metabolism, but may determine CTL function. Moreover, in another recent study of memory T cells, inhibition of AKT or PI3K signaling prevented Th17 cytokines being expressed by gamma c cytokine stimulation (IL-2, IL-7, IL-15) [42]. These findings also suggest that Th17 cytokine production may be regulated in part by PI3K/AKT signaling. 

## 3 GENERAL IMPLICATIONS FOR DEREGULATED AKT/MTOR SIGNALING

### 3.1 Balance Between Isoforms

Although the AKT isoforms are similarly activated by upstream signaling events and exhibit similar kinase functions, the ablation of individual isoforms in mice reveals distinct physiologic roles. An early study showed that the AKT1 isoform is required for normal growth, but dispensable for glucose homeostasis [43]. Moreover, severe diabetes, age-dependent loss of adipose tissue, and mild growth deficiency were observed in mice lacking AKT2 [44]. AKT3 knockout mice exhibited brain abnormalities [45]. These phenotypes may be attributed to tissue-specific expression of the isozymes and perhaps isoform-specific substrates. 

There are many studies that show that different AKT kinases may not completely overlap in function, and that isoform-specific signaling contributes to the diversity of intracellular signaling effects, as previously reviewed [46]. The role of AKT isoforms in the regulation of glucose homeostasis is one of the best-characterized AKT-mediated processes. However, tissue-specific isoform expression and activation do not explain all of the findings in different contexts. There is rationale for delineating AKT isoform-specific binding partners that regulate subcellular location and specific substrate recognition, for which information is lacking. Regulation of metabolism and cancer by AKT have been researched extensively, yet there is still limited knowledge regarding how the AKT family members regulate or crosstalk with other intracellular signaling molecules to mediate these two processes. Understanding the specific roles of AKT family members and the molecular mechanisms that determine AKT isoform functional specificity is still necessary in order to elucidate AKT regulated cellular processes and how AKT isoform-specific deregulation might contribute to disease states. 

Relatively little is known about the roles of individual isoforms of AKT in glucose homeostasis *in vivo*. A recent review summarizes data on the role of AKT isoforms in glucose homeostasis and diabetes by examining the phenotypes of different combinations of AKT isoform knockout and heterozygous gene combinations [47]. Overall results suggest that AKT1 can compensate for an AKT2 deficiency with regard to glucose homeostasis and diabetes, and that AKT isoforms may have complementary and compensatory roles in glucose homeostasis *in vivo*. Haplodeficiency of AKT1 in mice within an AKT2 null background can convert a pre-diabetic phenotype to overt type 2 diabetes, which is reversible by haplodeficiency of PTEN [48], whereas AKT3 does not appear to contribute significantly to diabetes. AKT1+/-, AKT2-/- mice exhibited hyperglycemia due to β-cell dysfunction and impaired glucose homeostasis was attributed decreased leptin levels [48]. β-cell dysfunction and impaired insulin secretion also was attributed to diminished insulin and glucose transporter 2 (GLUT2) protein expression due to reduced mTORC1 activity and mRNA translation in the islets, although the mechanism is unknown and may be mediated by the lack of a positive feedback through the insulin receptor that requires IRS-1 and IRS-2, as well as mTORC1 activity. AKT activity may be required for the positive feedback to stimulate insulin synthesis in these mouse models. 

Contrary to a previous report that the AKT1 isoform is required for normal growth but dispensable for glucose homeostasis [43], a recent study proposed a novel role for AKT1 in the regulation of glucose homeostasis, in that AKT1 null mice displayed an opposite phenotype of AKT2 null mice with improved insulin sensitivity, lower blood glucose, and higher serum glucagon concentrations [49]. It was confirmed that AKT2 null mice are insulin resistant and glucose intolerant with compensatory increase of β-cell mass, while AKT1 null mice had lower blood glucose levels, were more insulin sensitive, and exhibited higher serum glucagon concentrations accompanied by a mild increase in β-cell mass and proliferation. Additionally, signaling analyses revealed that AKT1, but not AKT2 or AKT3, is specifically activated by overexpression of IRS2 in β-cells and required for IRS2 action in the islets. Thus, there is still a need for improved understanding of the specific activities of each isoform in normal and disease tissues in order to clarify the context in which these AKT isoforms are important. 

Aside from glucose homeostasis and insulin signaling, a recent study reported nonredundant functions for AKT isoforms in the growth of murine astrocytes containing mutations of EGFR, p53, and/or PTEN and in human gliomas [50]. Loss of AKT1 or AKT2 decreased the proliferation of PTEN wild-type astrocytes, but loss of multiple isoforms was required to inhibit the proliferation of PTEN-null astrocytes. In contrast, AKT3 was required for anchorage-independent growth of transformed astrocytes and human glioma cells, and AKT3 loss inhibited invasion of transformed astrocytes. Furthermore, tumorigenesis of PTEN; p53-null astrocytes expressing the EGFRvIII mutation was inhibited by AKT1 loss, but accelerated by AKT2 loss. The finding that different AKT isoforms have different affects on the tumorigenic process is consistent with other tumor models that show differences between the signaling of AKT isoforms in tumorigenicity [51, 52]. 

### 3.2 AKT/mTOR Deregulation in Diabetes and Insulin Response

The role of AKT is implicated in human type 2 diabetes with the identification of a family with type 2 diabetes that had inherited loss of AKT2 missense mutation that seems to act in a dominant-negative manner to inhibit other AKT isoforms [53]. A subsequent analysis showed that heterozygous loss-of-function mutations in AKT2 can cause a syndrome of severe insulin resistance and lipodystrophy in humans, but that such mutations are rare [54].

Recently, a 12-kb haplotype upstream of the human AKT1 gene also was found to be associated with insulin resistance [55]. A genotype/phenotype association was found with fasting glucose and insulin levels as well as predisposition for the development of metabolic syndrome in human populations [56]. Overall, transcriptional regulation of AKT1 protein expression was suggested to be very complex, with multiple enhancers and repressors showing cell- and differentiation-specific effects. Polymorphisms of three regions seem to have co-evolved to establish a haplotype that may serve to coordinate metabolic phenotypes of fasting glucose, insulin, body mass index (BMI) and predisposition to metabolic syndrome. 

Mouse models have been extremely valuable in determining the role of AKT/mTOR in normal pancreatic islet cell physiology and function. In fact, there are a number of recent reports described below that implicate not just AKT/mTOR, but numerous other downstream signaling effectors in the regulation of β-cell function and *in vivo* insulin response.

Evidence suggests that AKT and downstream intracellular signaling play an important role in insulin resistance and the ability of β-cells to adapt to increased demand for insulin [57]. β-cell mass is controlled by various processes such as cell proliferation, survival/apoptosis, and cell size, and AKT activates several signaling components including FOXO, GSK-3, TSC2/mTOR and cell-cycle components that are involved in these processes. Each component is thought to contribute to the overall phenotype. Several mouse models exist with varying phenotypes affecting β-cell mass and plasticity, including PTEN targeted lines [58-61]. Although the importance of AKT and PTEN in the regulation of β-cell differentiation and neogenesis has been addressed, there is still a need to define the importance of AKT signaling in pancreas development, differentiation and cellular plasticity.

When AKT2 knockout mice were mated with mice deficient in serum- and glucocorticoid-regulated kinase 3 (SGK3) gene, an unexpected role was observed for SGK3 in islet β-cell function [62]. Double-knockout mice had worse glucose homeostasis than AKT2 null mice, including higher baseline glucose and increased blood glucose after glucose challenge. Double-knockout mice had lower plasma insulin and C-peptide levels, lower β-cell mass, reduced glucose-stimulated insulin secretion, and greater sensitivity to exogenous insulin than AKT2 single nulls. In addition, β-catenin expression was lower in the islets of double-knockout mice. These data provide *in vivo *evidence of a physiological role of SGK3 in β-cell function that may be associated the expression and activity of β-catenin, a mechanism that needs clarification.

Rictor/mTORC2 also was found to be important for maintaining β-cell proliferation and cell size, since Rictor null mice exhibited mild hyperglycemia and glucose intolerance caused by a decreased β-cell mass, β-cell proliferation, pancreatic insulin content, and glucose-stimulated insulin secretion [63]. Islets exhibited decreased AKT Ser473 phosphorylation and increased expression of FOXO1 and p27 proteins. Islet cells were not the only insulin-responsive tissue affected, as loss of Rictor in adipocytes prevented insulin-stimulated phosphorylation of AKT, and impaired the phosphorylation of downstream targets such as FOXO3a and AS160, but not GSK-3β phosphorylation at Ser9 [64]. Furthermore, Rictor null adipocytes exhibited impaired insulin-stimulated glucose transporter 4 (GLUT4) translocation and decreased glucose transport. The Rictor mouse model demonstrated the role for mTORC2 in the regulation cell function, whole-body glucose levels and insulin sensitivity.

Another recent study implicated mTORC1 and downstream S6K1 in a negative feedback mechanism involving insulin-induced glucose uptake in adipocytes [65]. Inhibition of mTORC1 by rapamycin or knockdown of mTOR, Raptor, or S6K1 reduced serine phosphorylation of IRS-1, but increased its insulin-stimulated tyrosine phosphorylation and associated PI3K activity. Following the disruption of the mTORC1/S6K1 pathway, insulin-stimulated activation of AKT2 and expression of GLUT4 were diminished, thereby impairing insulin-mediated glucose uptake despite increased PI3K activation. Overall, it was suggested that inhibition of mTORC1/S6K1 uncouples IRS1/PI3K signaling from insulin-induced glucose transport due to impaired activation of AKT2 and diminished expression of glucose transport. These findings help explain previous observations of impaired glucose homeostasis in rapamycin-treated subjects.

Mouse models also demonstrated a crosstalk between S6K1 and AKT1 using mice that overexpressed the constitutively active form of AKT1 under the rat insulin promoter [66]. RIP-MyrAKT1 mice exhibited enlarged β -cells, high plasma insulin levels, and improved glucose tolerance. Moreover, a subset of mice developed insulinomas with aging that contributed to decreased survival. Interbreeding of RIP-MyrAKT1 and S6K1-deficient mice resulted in reduced insulinemia and glycemia, and improved insulin secretion and sensitivity in mice with both defects compared to RIP-MyrAKT1 mice. Hyperplastic transformation was required for S6K1, although the increase in β-cell size in RIP-MyrAKT1 mice was not affected by S6K1 deficiency and may be influenced by other intracellular factors. Overall, the findings highlight S6K1 as a critical element for MyrAKT1-induced tumor formation in the context of pancreatic islet cells.

### 3.3 Role AKT/mTOR Hyperactivation in Cancer 

AKT/mTOR signaling is known to be important for several hallmark cellular processes that give cancer cells a competitive advantage over that of normal cells. Specifically, alterations in several integral players of the AKT/mTOR signaling pathway can be classified as somatic mutations that activate additional downstream pathways or disruptions of negative-feedback mechanisms that attenuate proliferative signaling, as has been extensively reviewed [67]. Moreover, the role of AKT in regulating apoptosis signaling and the role of mTOR in autophagy are clearly important for decisions regarding cell survival and death. 

Several reports beyond the scope of this review substantiate the important role of AKT/mTOR in cancer through genetic studies in patient populations, through the use of mouse models to dissect out the role of the pathway in specific aspects of cancer and by continued experimentation on cancer cell lines to delineate mechanistic impacts. Overall, these studies support the importance of finding therapeutic avenues to address AKT/mTOR hyperactivation.

Several permutations of oncogenes and tumor suppressor genes along the AKT/mTOR pathway have been summarized extensively, as exemplified by a prior review [68]. In general, intrinsic activation may be through either mutation of the PI3K p110 catalytic subunit (leading to permanent activation) or mutation of the PI3K p85 regulatory subunit (relieving its inhibition on the p110 subunit), by gene amplification or protein overexpression of AKT, or by loss of regulatory inhibition of TSC proteins or PTEN function (either through promoter methylation, gene mutation, or allelic deletion) [69]. 

An increasing number of studies have implicated short nucleotide polymorphisms (SNPs) in several pathway-specific components that may influence genetic predisposition or susceptibility to cancer or other diseases. Among these recent studies, SNPs in genes encoding various signaling components along the PI3K-PTEN-AKT-TSC-mTOR-S6K signaling pathway were found to be associated with colon and rectal cancer risk [70, 71] or disease progression in non-small cell lung cancer (NSCLC) patients treated with platinum compounds [72]. Moreover, high caloric intake and low physical activity was associated with increased bladder cancer risk, which increased several fold in individuals with multiple polymorphisms in genes encoding proteins involved in the mTOR pathway, including Raptor [73].

In a recent tumorigenicity study, expression of mTOR, Raptor, and Rictor mRNAs were associated with advanced-staged colorectal cancer. Protein levels also were significantly elevated in stage IV primary colorectal tumors and their matched distant metastases, relative to that of normal colon [74]. Rapamycin treatment or stable inhibition of mTORC1 (Raptor) and mTORC2 (Rictor), inhibited migration, invasion, and *in vivo* metastasis, and was associated with alterations of the cytoskeleton and decreased activation of RhoA and Rac1. Knockdown of mTORC1 and mTORC2 induced a mesenchymal–epithelial transition (MET) and enhanced chemosensitivity of colorectal cancer cells to oxaliplatin. Therefore, colorectal cancer patients may benefit from new dual mTOR kinase inhibitors that target the ATP binding pocket to inhibit both mTORC1 and mTORC2 as part of a therapeutic strategy. 

The discovery of TSC2 as a negative regulator of mTORC1 was a major advancement in the understanding of tuberous sclerosis (TS) and pulmonary lymphangioleiomyomatosis (LAM) [75]. Through the formation of a complex between TSC1/TSC2, these tumor suppressors control the activity of the small GTPase Rheb (Fig. **1**). Mutational inactivation of TSC2 led to constitutively active mTORC1, increased cell proliferation and pathological changes consistent with TS and LAM. Recently it was shown that mTORC2 mediates TSC2-null cell proliferation and survival through RhoA GTPase and Bcl2, and that proliferation was inhibited by re-expression of TSC2, or inhibition of Rheb, mTOR, Raptor or Rictor [75]. siRNA against Rictor inhibited RhoA GTPase activity and AKT Ser473 phosphorylation, whereas constitutively active V14RhoA rescued growth inhibition. In summary, the data showed that mTORC2-dependent activation of RhoA is required for TSC2-null cell growth and survival, and that targeting both mTORC2 and mTORC1 should be a promising strategy in diseases with TSC2 dysfunction. 

mTORC2 also was shown to regulate cancer cell migration through selective activation of AKT1 [76]. IGF-1 induced SKOV-3 ovarian tumor cell migration was abolished by the PI3K inhibitor LY294002 or by AKT inhibitors, whereas inhibition of ERK or mTORC1 did not affect IGF-1- induced SKOV-3 cell migration. Silencing of Rictor also abolished IGF-1-induced ovarian tumor cell migration and phosphorylation of AKT, although the inactivation of mTORC1 by silencing of Raptor had no effect. Rictor was associated more with AKT1 rather than AKT2, and overexpression of Rictor facilitated IGF-1-induced AKT1 activation, whereas silencing of AKT1 but not AKT2 inhibited IGF-1- induced ovarian tumor cell migration. Moreover, P-Rex1 had an important role in the activation of AKT1 and in mTORC2/AKT1 signaling, thereby contributing to cancer cell migration. There may be a positive feedback loop between AKT1 and P-Rex1/Rac1, since knockdown of P-Rex1 attenuated AKT activation as well as IGF-1-induced SKOV-3 cell migration and silencing of AKT1 inhibited IGF-1-induced P-Rex1 translocation to regions of membrane-ruffling.

## 4 IMPLICATIONS FOR RESET OR REPROGRAMMED AKT/MTOR SIGNALING 

### 4.1 Molecular Inhibitors to Target AKT/mTOR

Many small molecule inhibitors are cytostatic rather than cytotoxic, so they may need to be combined with a therapeutic modality that induces cell death. More than one signal transduction pathway may need to be targeted in order to eradicate tumor growth, or to prevent drug resistance and the recurrence of cancer initiating cells.

As discussed in a recent review, various signal transduction inhibitors have been evaluated as radiosensitizers [77]. Moreover, mTOR and radiation play critical roles in the regulation of autophagy. There is an increase in autophagy when mTOR is blocked by rapamycin. Cancers containing PIK3CA mutations are often sensitive to rapamycin and its analogs (rapalogs). However, PIK3CA mutant cells may have additional mutations of KRAS that contribute to resistance to rapalogs, perhaps due to complicated feedback loops between the RAS/Raf/MEK/ERK and PI3K/PTEN/AKT/mTOR pathways. Also, these pathways may be further activated by chemotherapeutic drugs and ionizing radiation, which may contribute to the emergence of drug resistant clones. To interfere with cancer progression or prevent aging, it may be necessary to target both the RAS/Raf/MEK/ERK and PI3K/PTEN/AKT/mTOR signaling pathways to induce apoptosis. A review by Chappell *et al.* details many of the small molecule inhibitors that are available for the targeting of Raf/MEK and PI3K/PDK/AKT/mTOR pathways [77]. 

Another recent review by McCubrey *et al*. discusses in detail how PTEN regulation is an example of a tumor suppressor gene in the AKT pathway that is frequently deregulated in human cancer and associated with resistance to therapy [78]. Activation of the PTEN/AKT/mTOR pathway may result in increased transcription of many genes that promote cell growth and malignant transformation. Genetic screening prior to treatment with inhibitors targeting the RAS/Raf/MEK/ERK and PI3K/PTEN/AKT/mTOR pathways may be an important determinant for molecular dependence or even oncogene addiction to the signaling pathway. Various components of the PI3K/PTEN/AKT/mTOR pathway are implicated in drug resistance, and changes in AKT activity may occur as a result of mutations in PI3K or PTEN. Furthermore, altered expression of miRNAs may be involved in decreasing PTEN expression, which results in drug resistance.

**Fig. (4):**
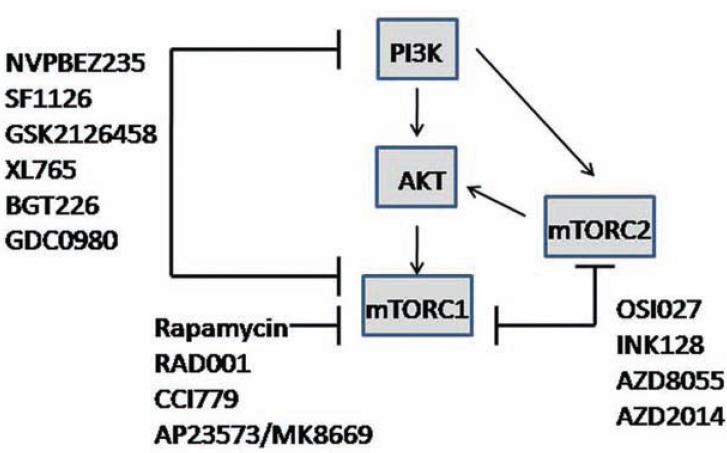
General diagram of rapalogs, ATP-competitive mTOR inhibitors and dual AKT/mTOR pathway inhibitors that are clinical use. Primary source was Zhang *et al.* [80].

Specific to mTOR, rapamycin and its rapalogs temsirolimus (CCI779, Wyeth), everolimus (RAD001, Novartis), and ridaforolimus (AP23573/MK-8669, ARIAD-Merck) (Fig. **4**) are known to be effective against renal cell carcinoma, and are being investigated for the treatment of other malignancies [79]. However, rapalogs are known to have drug resistance difficulties that may ultimately limit their utility as single agents in therapy. Sensitive tumors may be responsive to low doses of rapalogs, but there is insufficient ability to predict response other than through potential dependence on the hyperactive AKT/mTOR pathway. Higher doses or rapalogs given concurrently with other targeted therapeutic agents may be required for therapeutic efficacy in patients that may have tumors with redundant signaling pathways that interfere with rapalog efficacy. Future studies are needed to establish molecular profiles or biomarkers that predict sensitivity to mTOR inhibitor therapy. 

The identification of tumors that respond to mTOR inhibitors is still a major problem and has been highlighted in several recent reviews [69, 79]. Activation of the PI3K/AKT/mTOR pathway in itself has not been sufficient to predict sensitivity. Besides oncogenic KRAS expression which may complicate response by activating RAS/Raf/MEK/ERK signaling, potential mechanisms that may lead to resistance to mTOR inhibition include possible mutations in FKBP-12 that diminish the affinity for rapalog binding or the FKB domain of mTOR, or incomplete suppression of downstream targets such as 4E-BP1 phosphorylation [79]. Another complicating factor in tumor cells may be a non-functional apoptotic pathway, especially when Bcl2 is overexpressed and there is resistance to apoptosis or alternative survival pathways [69]. Moreover, clinical findings suggest that malignant tumors appropriate for mTOR inhibitors may include those that are highly addicted to angiogenesis (such as renal cell carcinoma, neuroendocrine tumors and possibly hepatocellular carcinoma) or to cyclin D1 overexpression (such as mantle cell lymphoma). 

The rationale for using dual targeting inhibitors such as PI3K/mTORC1 or ATP-competitive mTORC1/mTORC2 inhibitors (Fig. **4**) is that hyperactive PI3K/AKT/mTOR signaling is prevalent in a broad spectrum of human cancers; treatment of tumor cells with first generation rapalogs unexpectedly resulted in the upregulation of phosphorylated AKT and may potentially circumvent the effectiveness of targeting the pathway; and new evidence suggests that mTORC2, which is involved in the rephosphorylation of AKT, is also involved in cancer cell growth and survival [80]. Overall, dual PI3K/mTORC1 or mTORC1/mTORC2 inhibitors appear to work better than targeting mTORC1 alone at inhibiting the signaling pathway and blocking re-phosphorylation of AKT. Although the data are still being collected, several inhibitors are in early-stage clinical trials and have promising results. A review by Zhang *et al*. describes many of these inhibitors, their chemical structure, specificity, route of administration and overall status in clinical development [80]. For example, the dual PI3K/mTOR inhibitor NVP-BEZ235 (Novartis) is an orally bioavailable reagent that was reported to inhibit tumor growth in many preclinical models, and it enhanced the antitumor activity of several other cancer drugs. Important for understanding mTOR signaling and inhibition beyond that of rapamycin, a new generation of mTOR-specific inhibitors for the kinase active site of mTOR has emerged. This class of inhibitors is important because they block the activity of both mTOR complexes, and among them, INK128, AZD8055, OSI027 and AZD2014 have already entered clinical trials (Fig. **4**).

As a strategy to induce the maximal inhibition of this pathway in cancer cells, allosteric mTOR rapalogs were combined with a proof of principle dual PI3K/mTOR kinase inhibitor (PI-103) and shown to exhibit more activity than single agents in human ovarian and prostate cancer cells with alterations in the pathway [81]. Combined inhibition of mTOR prevented activation of AKT and caused more sustained inhibition of AKT phosphorylation relative to rapamycin or RAD001 treatment. The combination also inhibited the expression of downstream proteins and 4E-BP1 phosphorylation to block CAP-dependent translation. Cell cycle arrest at the G1 phase was more sustained when cells were treated with the combination treatment. Moreover, a proteomic approach identified MYC as a mediator of decreased downstream proteins affected by the combination treatment targeting mTOR. Overall, the combination of an allosteric rapalog and a catalytic inhibitor of mTOR showed more effectiveness, without increased toxicity, compared to single drug alone, and this approach may have therapeutic implications in a clinical setting.

The dual PI3K/mTOR inhibitor PI-103 was found to induce autophagy in therapy resistant glioma [82] Inhibitors of autophagosome maturation cooperated with PI-103 to induce apoptosis through the mitochondrial pathway. Dual mTORC1/mTORC2 inhibition stimulated more autophagy relative to rapamycin, without activating AKT, and resulted in increased glioma cell apoptosis when combined with a drug inhibition of autophagy. Moreover, the PI3K-mTOR inhibitor NVP-BEZ235 also synergized with chloroquine, an inhibitor of autophagy, to induce apoptosis in glioma xenografts *in vivo*. 

In another study, the dual mTORC1/mTORC2 inhibitor AZD8055 (AstraZeneca) was shown to potentiate chemotherapy-mediated autophagy, as shown by LC3I-II conversion and down-regulation of the ubiquitin-binding protein p62/sequestosome 1 [83]. AZD8055-induced autophagy contributed to survival in response to cytotoxic chemotherapeutic drugs, but inhibition of autophagy partially reversed the protective effect of AZD8055. Knockdown of mTOR, but not Raptor or Rictor, reduced the phosphorylation of ULK1 and enhanced chemotherapy-induced autophagy to cause a similar pro-survival effect relative to AZD8055. These investigations, along with that of the rapalog/PI-103 combinations in ovarian and prostate cancers and previous use of dual PI3K/mTOR inhibitor PI-103 in glioma cells, collectively demonstrate the effectiveness of targeting more than one component of the AKT/mTOR pathway, although the role of autophagy in chemotherapeutic response still needs to be delineated.

### 4.2 Nutrient/Calorie Restriction and Control of mTOR Signaling for Longevity

As discussed above, a main function of mitochondria is to synthesize ATP via oxidative phosphorylation or OXPHOS. Mitochondria produce reactive oxygen species (ROS) that must be detoxified as a side product of normal respiration, or successive replications of mitochondria may accumulate mutations that eventually compromise the efficiency of OXPHOS to contribute to inefficiencies or even toxicity as cells age, as summarized previously [84]. Mitophagy eliminates dysfunctional or damaged mitochondria. Decreased expression of genes that regulate autophagy or mitophagy can cause degenerative diseases. Thus, a combination of mitochondrial dysfunction and inadequate autophagy may contribute to aging-associated pathologies. 

**Fig. (5):**
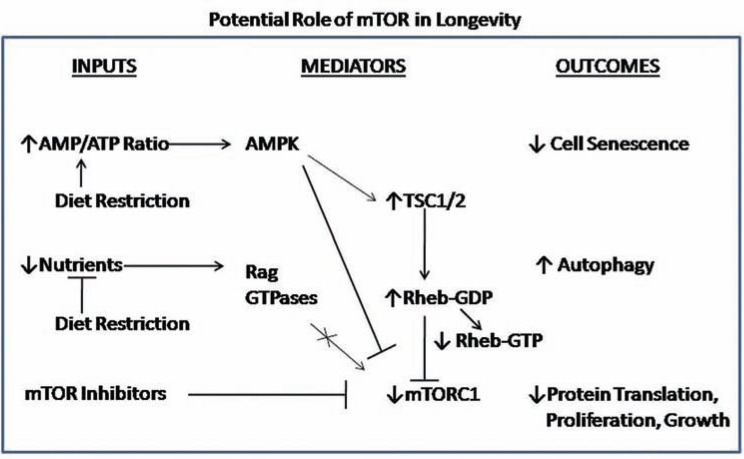
Schematic diagram of how mTOR may play a role in longevity. Calorie or dietary restriction is postulated to increase the AMP/ATP ratio as a result of diminished energy production and decreased nutrient availability. mTOR inhibitors such as rapamycin may be considered mimics for calorie or dietary restriction because ultimately they reduce mTORC1 activity, similar to the affect of AMPK on inhibitory mechanisms to regulate mTORC1, and inhibition by nutrient deprivation of the Rag GTPases, which would normally stimulate mTORC1. As a consequence of diminished mTORC1 activity, protein translation, proliferation and overall growth would be limited and the dynamics of decreased mTORC1 would favor increased autophagy to sustain cell survival and prevent cell senescence.

mTORC1 integrates cellular response to stress and nutrients, and responds by altering cellular metabolic processes to adapt to nutrient supply and maintain whole-body metabolic homeostasis (Fig. **5**). Defects in mTORC1 regulation, arising from either physiological or genetic conditions, may contribute to the metabolic dysfunction underlying a variety of human diseases, including type 2 diabetes [85]. There is a need to understand the functions of mTORC1 in regulating the physiological state of mammals. Gain- and loss-of-function models have been developed to delineate the role of mTORC1 signaling in rodents, but null alleles of mTOR or Raptor or upstream TSC1/2 result in early embryonic lethality. Alternatively, rapamycin is used as a tool to understand the physiological functions of mTORC1. Although different rodent models have yielded variable results in experimental efforts to control glucose and insulin levels, rapamycin has been shown to block weight gain and decrease fat. A recent review by Howell *et al.* provides a detailed table summarizing the metabolic phenotypes resulting from perturbation of mTORC1 in metabolic tissues [85].

Aging is often associated with a reduced autophagic potential, whereas life-prolonging strategies often stimulate autophagy [86]. Longevity can be extended through pharmacological or genetic manipulations that stimulate autophagy, and also caloric restriction (CR), inhibition of insulin/insulin growth factor signaling, rapamycin, and other treatments. A recent review by Rubinsztein *et al.* details aging-related phenotypes of autophagy-deficient mice and the associations between autophagy and anti-aging strategies [86].

However, clarification is needed regarding whether the effect of the rapamycin or CR on longevity is mediated through autophagy or alternative pathways. Stimulating autophagy at a mature age also has shown beneficial effects on aging in mice [86]. CR does not further increase life span when TOR signaling is already reduced in yeast, worms, or flies, so mechanisms common to both interventions may mediate these anti-aging strategies. Inhibition of TOR, either pharmacologically (with rapamycin) or genetically, has been shown to extend life span in yeast, C. elegans [87], D. melanogaster [88], and mice [89]. Rapamycin also may impact aging of mice through its capacity to suppress inflammatory and autoimmune processes [90] that negatively affect longevity, or through its effects on protein translation. 

Inhibiting mTOR signaling slows aging by affecting a number of downstream processes including mRNA translation, autophagy, endoplasmic reticulum stress, stress responses and metabolism, as summarized recently [90]. The phenotypic overlap of dietary restriction and reduced translational initiation suggests that inhibition of protein synthesis is likely to be important for lifespan extension. For example, inhibition of mTORC1 or S6K inhibits growth, but unlike mTORC1, S6K is not essential for development. Identifying mechanisms by which the mTOR signaling delays aging may provide therapeutic strategies for age related diseases to facilitate healthful longevity.

Calorie restriction (25–40% below ad libitum intake) can improve insulin sensitivity, since CR–induced, insulin-stimulated 2-deoxyglucose (2DG) uptake was greater in isolated skeletal muscles from wild type compared with AKT2 knockout mice after 20 days of CR vs. ad libitum feeding [91]. CR increased AKT2 Ser473 phosphorylation greater than that of ad libitum levels found in insulin-stimulated WT mice, and AKT1 phosphorylation was insufficient for insulin stimulated glucose uptake in AKT2-deficient muscles. Thus, it was concluded that AKT2 is crucial for the full effect of CR on glucose uptake in skeletal muscle.

Diet induced changes to circulating IGF-1 to modulate IGF-1R/EGFR activation and signaling downstream of AKT and mTOR [92]. Moreover, CR affected cell cycle regulatory proteins, thereby regulating proliferation, and modulating susceptibility to tumor development. As recently summarized [93], dietary restriction also was shown to be effective in extending the lifespan of p53-deficient mice, in reducing intestinal polyps of ApcMin mice and in a lung cancer model with intact PI3K signaling, but had minimal effects on retinoblastoma (Rb)-deficient neuroendocrine tumors. Targeting mTORC1 may be a CR mimetic strategy that potentially inhibits tumorigenesis. Since growth factor (e.g., IGF-I) and nutrient (e.g., amino acids) reductions extend lifespan and reduce cancer, and mTOR integrates responses, it was proposed that mTOR is central to the aging process and its chronic inhibition might delay or reduce the severity of cancer [93]. 

Rapamycin also was found to mimic the effects of CR on Panc02 murine pancreatic cancer cells orthotopically implanted in C57BL/6 mice [94]. In contrast to rapamycin, CR relative to ad libitum-fed control diet reduced body weight, improved glucose responsiveness, and decreased circulating levels of insulin-like growth factor (IGF)-1 and leptin. Pancreatic tumor volume and proliferation was decreased in both groups, although to a greater extent in the CR group. Phosphorylation of mTOR, p70/S6K, and S6 ribosomal protein was decreased in both groups, but only CR decreased phosphorylation of AKT, GSK-3β, ERK/MAPK, and STAT3. These findings provided evidence that rapamycin partially mimics the anticancer effects of CR on tumor growth in a murine model of pancreatic cancer, but that CR had a stronger effect on global metabolism than treatment with low dose rapamycin.

### 4.3 Targeting AKT/mTOR to Reprogram Immune Cell Homeostasis

CD8 T-cell adaptive immunity response to an antigen consists of stages of expansion, contraction and memory, and it is increasingly evident that mTOR has a role in these aspects of T cell development, as reviewed elsewhere [95]. In general, naïve CD8 cells can differentiate into effector CD8 T cells and memory CD8 T cells, which can persist in low numbers and then are activated in response to re-stimulation by antigen. During expansion, antigen-stimulated naïve CD8 cells increase by ~10^5^, express cytokines and homing markers, and acquire cytotoxic or effector functions. The CD8 effector population contracts by 90-95% after clearance of foreign antigen and the remaining cells exhibit CD8 memory differentiation. 

MHC class I-antigen, co-stimulatory molecules and cytokines are needed for the full activation and differentiation into CD8 T cells, although the mechanism is still being delineated. CD8 T cells often work in association with CD4 helper cells, and recently it was demonstrated that conditional deletion of Rictor impaired differentiation into T helper 1 (Th1) and Th2 cells without diverting T helper cell differentiation from FoxP3 or Th17 expressing cells [96]. In response to T cell activation, mTORC2 also promoted phosphorylation of AKT and PKC, and nuclear NF-kB transcription factor. Constitutively active AKT rescued T-bet (T-box expressed in T cells) transcription factor expression and Th1 but not Th2 cell differentiation, whereas activated PKC-θ rescued expression of GATA3 transcription factor and the Th2 cell defect of mTORC2 mutant cells. Thus, it was concluded that there are different pathways by which mTORC2 regulates the development of Th1 and Th2 cell subsets through mTOR-AKT and mTOR-PKC, respectively. 

Alternatively, naïve CD8 T cells may undergo proliferation in the absence of antigen under conditions of lymphopenia (abnormally low level of lymphocytes in the blood) in response to self-antigen-MHC, interleukin-7 (IL-7), and/or IL-15 [97] The antigen-independent proliferation of naïve CD8 T cells is defined as homeostatic proliferation, and it plays an important role in maintaining normal CD8 T cell numbers. mTOR also has a central role in the homeostatic proliferation of CD8 T cell memory and anti-tumor immunity [97]. This supports the idea that the strength of antigen recognition and cytokine responsiveness are important determinants in the switching of memory cells to effector cells. Overall these studies and other described herein, provide the basis for the idea that mTOR inhibition and the enrichment of the CD8 population may work together to regulate T cell homeostasis. 

As previously indicated, cytokine stimulation plays a key role in naïve T cells differentiation and in the survival of CD8 effector and memory cells. AKT activity was shown to increase effector T-cell survival under conditions of depleted cytokine *in vitro*, but not during the contraction phase *in vivo* [40]. Constitutive AKT activity in CD8 T cells may induce a negative feedback mechanism that represses expression of IL-7 and IL-2/15 cytokine receptors and decreases signaling effectors such as phosphorylated STAT5 and Bcl2 expression [40]. Long-lived memory precursor cells induced with IL-15 activated AKT better than short lived effector cells, whereas constitutive STAT5 activity increased effector and memory CD8 T-cell survival and increased homeostatic proliferation, AKT activation, and Bcl2 expression.

The inverse relationship betweeen T-bet and Eomes transcription factors is the most widely studied gene expression signature that is characteristic of CD8 effector vs. CD8 memory cells, as summarized recently [98]. mTOR kinase is implicated in the balance of effector versus memory cell differentiation, in part through the regulation of these transcriptional factors (Fig. **3**). mTOR inhibition favored cell differentiation toward memory cells. Moreover, inhibition of mTOR activity by rapamycin inhibited T-bet expression and promoted memory precursor cells with improved tumor efficacy that may be attributed to alterations of cell phenotype, localization, survival and effector function upon antigen re-exposure. mTOR inhibition also may modulate the TCR signal strength so that there is not a robust activation of naïve CD8 cells toward effector cells, which are normally short lived [99]. In general, although these memory T cells may be weaker in their protective recall responses, the memory T cell subset may be a useful resource to exploit for *in vivo* host immunity to tumor and infection. 

A major focus in understanding mTOR/AKT signaling in immune cells is centered on its role in the cell survival. Although chemotherapy eliminates a majority of cancer cells, cancer stem cells may be left behind and they may be targeted for immunotherapy [100]. Recently it was found that the dual mTORC1/mTORC2 inhibitor AZD8055 enhances immunotherapy in part through CD40 [101], which is a co-stimulatory protein found on antigen presenting cells and required for their activation. In a renal carcinoma model, the combination of an αCD40 agonist, which is a mimic for the natural ligand CD154 to promote T cell response through activation of antigen presenting cells, and AZD8055 were synergistic at increasing the infiltration, activation and proliferation of CD8 T cells, but a synergistic interaction was not observed when rapamycin was substituted for AZD8055 [101]. 

Elimination of activated T cells through apoptosis also is a critical mechanism of immune homeostasis. IL-7 is known to be involved in the homeostatic proliferation and survival of peripheral T cells [102] although its signaling in human effector/memory T cells is still being defined. Previous studies on IL-7 signaling in mouse naïve T cells and IL-7-dependent T-cell clones indicated that IL-7 is connected to the JAK1/3-STAT5 and PI3K/AKT cell signaling pathways, and that IL-7-induced PI3K/AKT activation was implicated in cell survival mainly through the phosphorylation and inactivation of the Bcl-2 proapoptotic protein BAD and through glucose uptake, which is necessary to maintain T-cell viability [103]. IL-7 protected human CD4 effector/memory T cells from apoptosis induced through the absence of stimulation and cytokines, and IL-7 upregulated Bcl-2, Bcl-xL and Mcl-1, and induced activation of the JAK/STAT signaling pathway for cell survival and up-regulation of Bcl-2 proteins. IL-7 was found to be a weak activator of the PI3K/AKT pathway. Human effector/memory T cells express a basal level of phosphorylated AKT and treatment with a PI3K/ AKT inhibitor increased apoptosis, so PI3K/AKT can be important in maintaining basal cell viability.

Populations of T cells in human patients may display characteristics of senescent T cells normally associated with aging, but the link between senescence and tumor-induced T-cell suppression needs clarification. IL-7 may be important in protecting T cells from senescence [104]. IL-7 protected human T cells after a short co-culture with tumor cells *in vitro*, and inhibitors of PI3K/AKT pathway and siRNAs against Mcl-1 and Bim were used to evaluate the role of these signaling pathways in IL-7 protection. Overall, IL-7 inhibited CD27/CD28 loss and also maintained proliferative capacity, IL-2 production, and reduced suppressive function. The effect was mediated by the PI3K/AKT pathway, which inhibited downstream GSK-3β, preventing it from phosphorylating Mcl-1. Knockdown of Bim-1, a Mcl-1 binding partner and proapoptotic protein, protected T cells from dysfunctional alterations. These observations confirm the role for Bcl-2 family members in cytokine signaling and suggest that IL-7 treatment in combination with other immunotherapies may lead to new clinical strategies to maintain normal T-cell function and reduce tumor induced generation of dysfunctional and suppressor T cells.

While it is evident that mTOR signaling is involved in many aspects of T-cell biology, how mTOR complexes are regulated and overall importance to T cells is still being elucidated. Mature T cells circulate in the peripheral blood and lymphoid organs in a quiescent state (G0) characterized by small cell size and low metabolic activity. Deletion of TSC1 in murine T-cells led to exit from quiescence and apoptosis, which resulted in a reduction of the peripheral T-cell population [105, 106]. TSC1 was shown to establish quiescence in naïve T cells by controlling cell size, cell cycle entry and response to stimulation of the TCR. mTORC1 was constitutively activated in TSC1-deficient cells and exhibited highly phosphorylated S6K1, whereas mTORC2 signaling or AKT activity were decreased in TSC1-deficient T cells. Furthermore, TSC1-deficient T cells were found to have increased concentrations of reactive oxygen species (ROS) and exhibited decreased mitochondrial content and membrane potential, which was consistent with apoptotic signaling. Overall, results demonstrated that TSC1 differentially regulated mTORC1 and mTORC2 activity, promoted T-cell survival, and was critical for normal mitochondrial homeostasis in T cells.

## CONCLUDING REMARKS

There is an increasing amount of knowledge and reagents that are available for dissecting the role of AKT/mTOR in signal transduction cascades. It is known that the pathway is frequently hyperactivated in human cancers and efforts are underway to determine the best strategies to combat the deregulation. However, there still are many basic mechanistic questions regarding the role of AKT/mTOR in normal development and in homeostatic regulation of cell proliferation, differentiation, metabolism, trafficking and cell survival. Context and interplay between the AKT isoforms still need clarification. Tissue-specific or temporal contexts may affect protein expression, activity and crosstalk with downstream effectors. Pancreatic β-cells, insulin signaling and the contribution to diabetes and the balance of hematopoietic cells in immunity are the main examples that were illustrated in this review. 

mTOR is downstream of AKT and is known to be at the intersection of several cellular processes including cell cycle progression, anabolism and cell survival to control cell growth in response to nutrients, growth factors, energy and stress [14, 15]. The mechanism for inhibition of cell cycle progression and cell proliferation is still not clearly defined, although rapamycin has been shown to disrupt cyclin dependent kinase 4 (Cdk4)-cyclin D1 complexes and to upregulate the p27 Cdk inhibitor [107]. It also is a unique target because of its link to autophagy and its ties to mitochondrial function. Inhibition of mTOR through molecular targeted therapies or through nutrient deprivation may trigger this alternate pathway to enable cells to start to degrade cellular proteins and organelles to make amino acids available for alternate pathways of survival. 

Moreover, there is a link between calorie restriction or rapamycin and increased longevity in mice [94] but the specific mechanism regarding AKT/mTOR signaling effectors, the impact on cell energetics and the association with senescence or autophagy has yet to be defined. Deregulation of mTOR is linked to diabetes through excess food and energy intake, and weight gain which is linked to cardiovascular disease and other adverse health conditions [14, 15]. Thus, mTOR is an important molecular target for understanding control of cell homeostasis. It has been reported that inhibition of mTORC1 affects mitochondrial function through the inhibition of new mitochondria synthesis and promotion of a particular type of autophagy called mitophagy [84]. mTOR is activated when nutrients are available, but it is inactivated during fasting [14]. Suppression of mTOR activity is feasible using caloric or dietary restriction; mimetics; chemical inhibition of glycolysis and inhibition of mitochondrial function (ATP levels decrease to increase levels of AMP:ATP to activate AMP kinase) and analogs to rapamycin [90]. 

Finally, studies regarding AKT/mTOR signaling in immune cell homeostasis are yielding novel and unexpected perspectives on the importance of these signaling players in innate and adaptive immunity. Although the mTOR inhibitor rapamycin is primarily used as an immunosuppressant in transplant patients because it reduces B and T cell numbers, as well as for arresting tumor cell growth and progression, it is now evident that mTOR inhibition may actually favor increased numbers of certain subsets of immune cells that favor low mTOR activity and reduced mitochondrial energy expenditure. 

Collectively, it is expected that clarification of the roles of AKT/mTOR in the balance of cellular processes will contribute to our overall understanding of how and why deregulation of the pathway leads to disease and malignancy, while providing insights as to how and in what context to use inhibitors of AKT/mTOR for maximal effectiveness.
